# The impact of hydrocephalus on pediatric epilepsy: insights from a single-center investigation

**DOI:** 10.1186/s42494-025-00211-9

**Published:** 2025-04-07

**Authors:** Raidah Al-Baradie, AlAlwan Amena, Shahid Bashir

**Affiliations:** 1 Pedartic Neurology, Neuroscience Center, King Fahad Specialist Hospital Dammam, Dammam, 32253 Saudi Arabia; 2 Pediatrics Department, King Fahad Specialist Hospital Dammam, Dammam, 32253 Saudi Arabia; 3 King Salman Center for Disability Research, Riyadh, 11614 Saudi Arabia; 4 Pediatric Neurology, Neuroscience Center, King Fahad Specialist Hospital Dammam, P.O. Box 15215, Dammam, 31444 Saudi Arabia

**Keywords:** Hydrocephalus, Epilepsy, Pediatric neurology, Intracerebral hemorrhage, Shunt insertion, Seizure onset, Retrospective analysis

## Abstract

**Background:**

Hydrocephalus following intracerebral hemorrhage (ICH) is a common yet treatable complication. Despite its clinical significance, the long-term outcomes and predictive factors associated with hydrocephalus are not well understood, especially in pediatric population with epilepsy. This study aims to investigate the impact of hydrocephalus on children with epilepsy in Saudi Arabia.

**Methods:**

This observational retrospective study was conducted at the pediatric neurology clinic of a tertiary care epilepsy center. Twenty-six patients diagnosed with comorbid hydrocephalus and epilepsy were included. Key variables analyzed included seizure characteristics, age at seizure onset, use of antiseizure medications, seizure control, long-term developmental outcomes (intellectual and motor), the timing of shunt insertion in relation to seizure onset, and focal EEG changes.

**Results:**

Among the 26 patients, 61.5% (*n* = 16) were males. A notable proportion, 57.7% (*n* = 15), exhibited normal electroencephalogram (EEG) results, while 42.3% (*n* = 11) presented with abnormal EEG patterns. Of the 26 patients, 38.4% (*n* = 10) had congenital hydrocephalus, while 61.6% (*n* = 16) had acquired hydrocephalus. Seizure frequency varied: 38.5% (*n* = 10) experienced only once seizure, 11.5% (*n* = 3) had one to four seizures per month and 3.8% (*n* = 1) encountered two to four seizures per day. The majority of hydrocephalus cases (61.5%, *n* = 16) were diagnosed during infancy to two years of age. Shunt insertion was prevalent, with 80.7% (*n* = 21) undergoing the procedure. Developmental delays were observed in 61.5% (*n* = 16) of the patients.

**Conclusions:**

This study highlights the important role of hydrocephalus in the context of epilepsy among children in Saudi Arabia. Further research with larger sample size is needed to confirm these findings and provide a basis for improved understanding and targeted interventions in this critical medical area.

## Background

Hydrocephalus or a hydrocephalus-associated syndrome is linked to epilepsy, which is the second most prevalent neurological disorder [1, 2]. Genetic disorders associated with hydrocephalus have been reported in epilepsy [3]. In children, epilepsy is a common comorbidity, affecting 4.3 to 9.3 per 1000 children [4]. The etiology of epilepsy may be attributed to acute injury, elevated intracranial pressure, underlying brain abnormalities, or even shunt implantation [1, 3]. It is reported that approximately 30% of children with ventriculo-peritoneal shunts experience recurrent seizures [4].

In children, hydrocephalus is defined as an increase in intraventricular pressure leading to pathological dilatation of cerebral ventricles and the accumulation of cerebrospinal fluid (CSF). This condition disrupts the balance between CSF production, circulation, and absorption, resulting in varied pressure levels [3]. With an estimated incidence of six per 10,000 live births, hydrocephalus contributes to a 13% newborn mortality rate before initial hospitalization, establishing it as a significant cause of morbidity and mortality in the pediatric population [5–9]. The societal burden is substantial, evidenced by 38,200 to 39,900 hospital admissions, 391,000 to 433,000 inpatient days, and annual hospital expenditures ranging from $1.4 to $2.0 billion in the United States [4].

Although hydrocephalus can result from various congenital and acquired conditions, the prevalence of epilepsy among children undergoing shunting for hydrocephalus is higher than in the general population [7]. Contributing factors include the etiology of hydrocephalus, age at diagnosis or shunt placement, intracranial pressure, burr hole location, shunt revisions, time course after shunt insertion, use of antiseizure medications, and complications such as infection and shunt tube obstruction [1, 4, 10–14]. Despite numerous reports on these issues, conclusions remain varied and controversial [2].

This study delves into the identification of risk factors for seizures in patients with spontaneous intracerebral hemorrhage (ICH) and examines their functional outcomes. This retrospective study was conducted at a tertiary epilepsy center in Saudi Arabia over several years, aiming to offer insights into our initial experiences with hydrocephalus.

## Material and methods

### Study design

This retrospective cohort study enrolled 26 patients aged 0 to 18 years who were diagnosed with hydrocephalus and epilepsy at the pediatric neurology clinic. The study was carried out in compliance with international and local ethical guidelines, including the Declaration of Helsinki. This study was reviewed and approved by the local ethics committee of the King Fahad Specialist Hospital Dammam (approval number: NEU0318).

### Data collection

A systematic approach was used to gather a comprehensive dataset for this retrospective cohort study, covering various aspects of participants’ medical histories and clinical conditions. Demographic characteristics, such as age and sex, were recorded.

#### Seizure details

The seizure details were analyzed, which included a detailed description of seizure semiology, documenting the diverse manifestations and symptoms. The classification of seizures, frequency of seizures, and age of onset seizure provided valuable information regarding the chronicity and severity of the condition. Seizure terminology adhered to the definitions outlined by the International League Against Epilepsy (ILAE) [15].

#### Seizure treatment history

The exploration of history of the seizure treatment involved the recording of details related to antiseizure medications. This included the type, dosage, and the overall effectiveness of medications in controlling seizures, providing valuable insights into therapeutic management.

#### Neuroimaging findings

The structural aspects of the brain associated with hydrocephalus and epilepsy were thoroughly investigated. Up until June 2014, MRIs were conducted using a Signa Excite HDxt 1.5T unit (GE Healthcare). After that, a MAGNETOM Skyra 3T unit (Siemens Healthineers) was utilized. Both sets of MRIs followed to a standardized epilepsy protocol to maintain consistency in imaging procedures. Neuroimaging results were extracted from reports within the IMPAX medical imaging software (Agfa HealthCare), highlighting the dependence on a reliable and widely used platform for data retrieval.

### Statistical analysis

The data were collected and analyzed using the IBM SPSS for Windows, version 21 (IBM Corp., Armonk, NY, USA). All categorical variables are described in frequency and percentages; the mean and standard deviation were calculated for age at seizure onset.

## Results

### Demographics

Patient characteristics are summarized in Table 1. There was no significant difference in gender distribution, with 61.5% male (*n* = 16) and 38.5% female (*n* = 10) participants (*P* = 0.239). Age distribution ranged from 0 to 18 years, with significant differences observed among age groups (*P* < 0.001): 7.7% (*n* = 2) were aged 0–2 years, 19.2% (*n* = 5) were aged 3–5 years, and 73.1% (*n* = 19) were aged 6–18 years (Table 1). A significant difference was also observed in the distribution based on seizure onset age (*P* = 0.030), ranging from birth to 12 years (Table 1).

**Table 1:** Patient characteristics

Variable	N (%)	*P* Value
**Gender**		**0.239**
Male	16 (61.5%)	
Female	10 (38.5%)	
**Age Distribution**		< 0.001***
0–2 Years	2 (7.7%)	
3–5 Years	5 (19.2%)	
6–18 Years	19 (73.1%)	
**Age at Seizure Onset**		0.030*
0–2 Years	9 (34.6%)	
3–5 Years	1 (3.8%)	
6–18 Years	4 (15.4%)	
**Hydrocephalus Diagnosis Age**		0.006*
0–2 Years	16 (61.5%)	
3–5 Years	3 (11.5%)	
6–18 Years	7 (26.9%)	
**Hydrocephalus Type**		**0.239**
Congenital	10 (38.4%)	
Acquired	16 (61.6%)	
**Seizure Frequency**		0.004*
No frequency	12 (46.2%)	
Once	10 (38.4%)	
1–4 times/month	3 (11.5%)	
2–4 times/day	1 (3.8%)	
**Shunt Revisions**		**0.228**
1	7 (26.9%)	
2	8 (30.8%)	
3	4 (15.4%)	
4	2 (7.7%)	
**Developmental Outcome**		< 0.001***
Normal	5 (19.2%)	
Motor deficits	5 (19.2%)	
Intellectual deficits	1 (3.8%)	
Motor and Intellectual deficits	15 (57.7%)	

* *P* < 0.05

*** *P* < 0.001

### Type of seizures

Participants experienced generalized tonic-colonic seizures (15.4%, *n* = 4) and focal tonic and drop attack seizures (15.4%, *n* = 4). Seizure onset varied, with 34.6% (*n* = 9) occurring between birth and two years, 3.8% (*n* = 1) between three and five years, and 15.4% (*n* = 4) between six and 16 years. Seizure frequency varied: 46.2% (*n* = 12) experienced no seizures, 38.4% (*n* = 10) had once seizures, 11.5% (*n* = 3) had one to four seizures per month, and 3.8% (*n* = 1) had two to four seizures per day (*P* < 0.004, Table 1).

### Type of anti-seizure medications (ASMs)

Among the 11 patients (42.3%) receiving antiseizure medications, the majority (34.7%, *n* = 9) were on monotherapy. The remaining patients (7.7%, *n* = 2) were on two to five medications. Seizures were controlled in 34.6% (*n* = 9), while 7.7% (*n* = 2) had uncontrolled epilepsy.

### Neuroimaging

EEG results were normal in 57.7% (*n* = 15) of patients, with significant variation based on hydrocephalus type (congenital or acquired) All patients exhibited abnormal MRI findings, categorized as hemispheric (11.5%, *n* = 3), midline (23%, *n* = 6), posterior fossa (27%, *n* = 7), and ventricular system-related lesions (38%, *n* = 10).

### Developmental outcomes

Developmental delays were observed in 80.7% (*n* = 21) of patients. Intellectual disability was noted in 3.8% (*n* = 1), motor deficits in 19.2% (*n* = 5), 19.2% (*n* = 5) were developmentally normal, 57.7% (*n* = 15) had both motor and intellectual disabilities (Table 1).

### Hydrocephalus

Hydrocephalus was diagnosed between infancy and two years in 61.5% (*n* = 16) of patients, between three and five years in 11.5% (*n* = 3), and between six and 16 years in 26.9% (*n* = 7) (*P* = 0.006). Congenital hydrocephalus was observed in 38.4% (*n* = 10), while 61.6% (*n* = 16) had acquired hydrocephalus (Table 2). Shunt insertion was performed in 80.7% (*n* = 21) of patients, with 30.8% (*n* = 8) requiring two revisions (*P* = 0.228) (Table 1).

**Table 2:** Etiology of hydrocephalus

Condition	Frequency (*n*)	Percent (%)
Arnold-Chiari Malformation Type II^*^	3	11.5
Arnold-Chiari Malformation Type III^*^	1	3.8
Joubert Syndrome and Dandy-Walker Syndrome^*^	1	3.8
Congenital Hydrocephalus with Intraventricular Hemorrhage^*^	1	3.8
Dandy-Walker Syndrome^*^	3	11.5
Myelomeningocele^*^	1	3.8
Atypical Teratoid/Rhabdoid Tumor (ATRT)	1	3.8
Craniopharyngioma	3	11.5
Pilocytic Astrocytoma	3	11.5
High-Grade Glioma	1	3.8
Hydrocephalus Secondary to Intraventricular Hemorrhage (IVH)	1	3.8
Left Cerebral Peduncle Cavernoma	1	3.8
Left Temporal Arachnoid Cyst	1	3.8
Medulloblastoma	2	7.7
Post-IVH Hydrocephalus	1	3.8
Suprasellar Astrocytoma	1	3.8
Suprasellar Glioma	1	3.8
**Total**	**26**	**100.0**

*Congenital

### Correlations

Analysis of correlations between seizure type and developmental outcomes revealed varying percentages of intellectual and motor deficits among patients without seizures and those with generalized tonic-colonic epilepsy. Additionally, participants with congenital hydrocephalus showed distinct seizure patterns compared to those with acquired hydrocephalus. Among patients with shunts, different revision frequencies were observed based on seizure status.

## Discussion

Numerous studies have investigated the association between epilepsy and hydrocephalus in children, with findings varying widely and reported prevalence rates ranging from 9 to 65% for epilepsy attributed to hydrocephalus [1, 5]. However, it is crucial to note that our study specifically focused on patients presenting with both epilepsy and hydrocephalus. From 2010 to 2020, our hospital records identified 26 such cases, shedding light on specific trends in this patient subgroup.

The predominance of hydrocephalus diagnoses in males, observed in 61.5% of our patients, aligns with some existing literature [16, 17]. Nevertheless, the lack of statistical significance emphasizes the complexity of this relationship. Notably, studies from Nigeria and Jordan reported a higher prevalence among females, introducing a noteworthy regional variability [18, 19].

Congenital hydrocephalus is frequently associated with genetic mutations or structural anomalies, such as Dandy-Walker syndrome, craniopharyngioma, and pilocytic astrocytoma, which often lead to early and more severe developmental challenges [20]. In contrast, acquired hydrocephalus, typically caused by infections or trauma, may present later and is less likely to be associated with global developmental deficits [21]. These distinctions suggest that future studies should stratify developmental outcomes based on the type of hydrocephalus to provide more personalized treatment insights.

In contrast to our findings, where 57.7% of patients exhibited normal EEGs, previous studies have reported abnormal EEGs in approximately 80% of cases with hydrocephalus and epilepsy, suggesting potential variability in the electroencephalographic profiles of these patients [22–24].

In the present study, a significant difference was observed in the distribution of seizure onset age, consistent with the findings reported by Wirrell et al. [25]. The highest prevalence of developmental and epileptic encephalopathies (DEE) occurred in children with seizure onset between 0 and 2 years (44.6%), while the lowest prevalence was observed between 12 and 18 years (0.7%) [25]. These findings highlight the critical period for early identification of severe epilepsy syndromes. The etiology also plays a significant role, with genetic factors being more prevalent in early-onset cases and structural abnormalities more common in later-onset cases. This is supported by a study by Specchio et al. on drug-resistant epilepsy in children, which found that the age of onset was significantly earlier in cases with genetic causes compared to those with structural or immunological causes [26]. These studies highlight the importance of considering the age at onset when evaluating potential underlying causes of epilepsy [25, 26]. It is notable that central nervous system maturation and age-specific susceptibilities influence the type and severity of seizures. For example, benign childhood epilepsy with centrotemporal spikes (Rolandic epilepsy) typically manifests between ages 3 and 13, peaking around 8–9 years [27]. Conversely, juvenile myoclonic epilepsy often presents between ages 12 and 18, with a mean onset at 15 years [28]. These age-specific patterns reflect the influence of neurodevelopmental factors on seizure onset. Our findings emphasize the necessity of age-specific diagnostic and therapeutic approaches in pediatric epilepsy. Recognizing the typical age ranges for various epilepsy syndromes can facilitate early diagnosis and the development of personalized treatment strategies, potentially improving patient outcomes.

Shunt insertions emerged as a significant aspect of our study, with 26.9% of patients undergoing one insertion, 30.8% undergoing two insertion, and 23.1% undergoing more than two insertions (Table 1). This high prevalence of shunt insertions may contribute to the observed epilepsy in our cohort, aligning with previous literature highlighting an increased incidence of seizures in children following shunt procedures [29]. The literature also suggests that epilepsy is more prevalent in shunt-treated patients compared to control groups, emphasizing the importance of prompt diagnosis and intervention for shunt malfunctions or infections to prevent potential brain damage [30].

Our findings are consistent with studies indicating a potential correlation between shunt infection and epilepsy, although the existing literature remains inconclusive on this association [31, 32]. Noteworthily, there is absent of consensus on whether seizure activity serves as an early indicator of shunt dysfunction, which further complicates the interpretation of our results [8, 33]. However, the significant proportion of participants (34.6%) experiencing seizure onset between 0 and 2 years of age echoes Faillace and Canady’s findings, suggesting a positive relationship between seizure onset and newly inserted shunts [6].

A critical limitation of our study is the relatively small sample size in comparison to previous investigations.

## Conclusions

This single-center pilot study explores the relationship between hydrocephalus and epilepsy in children. The findings underscore the importance of early diagnosis, particularly the age at seizure onset and hydrocephalus diagnosis, as important factors influencing developmental and seizure outcomes. Limitation need to be noted, as this is a single-center study with a relatively small sample size. However, these results provide insights into the broader interplay between congenital and acquired hydrocephalus, seizure frequency, antiseizure medication treatment, shunt history, and motor and intellectual deficits. A larger sample size and multi-center studies are needed to achieve a more comprehensive understanding. Such studies would contribute to evidence-based interventions for children with hydrocephalus and epilepsy, considering the factors elucidated here and their long-term implications.

## Data Availability

All data presented in this draft are included within the manuscript.

## Abbreviations

ASM: Anti-seizure medication
CSF: Cerebrospinal fluid
DEE: Developmental and epileptic encephalopathies
EEG: Electroencephalogram
ICH: Intracerebral hemorrhage
ILAE: International League Against Epilepsy
